# Predictions of Oncotype DX^®^ High-Risk Classification Using Magnetic Resonance Imaging-Based Intratumoral Heterogeneity

**DOI:** 10.3390/bioengineering13060611

**Published:** 2026-05-24

**Authors:** Sung Joon Park, Won Hwa Kim, Jaeil Kim, Taewoo Kang, Ji-Young Park, Byeongju Kang, Joon Suk Moon, Ho Yong Park, Hye Jung Kim, Jeeyeon Lee

**Affiliations:** 1 School of Computer Science and Engineering, Kyungpook National University, Daegu 41566, Republic of Korea; sjp611@gmail.com (S.J.P.); threeyears@gmail.com (J.K.); 2 BeamWorks Inc., Daegu 41566, Republic of Korea; 3 Department of Radiology, School of Medicine, Kyungpook National University, Kyungpook National University Chilgok Hospital, Daegu 41404, Republic of Korea; greenoaktree9@gmail.com (W.H.K.); ant637@naver.com (H.J.K.); 4 Department of Surgery, Pusan National University School of Medicine, Busan 49241, Republic of Korea; taewoo.d.kang@gmail.com; 5 Biomedical Research Institute, Pusan National University Hospital, Busan 49241, Republic of Korea; 6 Department of Pathology, School of Medicine, Kyungpook National University, Kyungpook National University Chilgok Hospital, Daegu 41404, Republic of Korea; jyparkmd@knu.ac.kr; 7 Department of Surgery, School of Medicine, Kyungpook National University, Kyungpook National University Chilgok Hospital, Daegu 41404, Republic of Korea; libertas033@knu.ac.kr (B.K.); joonsukm@gmail.com (J.S.M.); phy123@knu.ac.kr (H.Y.P.)

**Keywords:** breast cancer, Oncotype DX^®^, habitat imaging, radiomics, dynamic contrast-enhanced magnetic resonance imaging, intratumoral heterogeneity, iTED

## Abstract

The Oncotype DX^®^ 21-gene recurrence score (RS) guides adjuvant chemotherapy decisions in estrogen receptor-positive, human epidermal growth factor receptor 2-negative (ER+/HER2−) breast cancer, yet requires invasive tissue sampling and involves substantial costs. This study evaluated intratumoral tumor ecological diversity (iTED), a habitat imaging approach, as a non-invasive complement for predicting Oncotype DX^®^ high-risk classification (RS > 25). This retrospective multi-center study included 312 patients with ER+/HER2− invasive breast cancer who underwent Oncotype DX^®^ testing (development: *n* = 168; external validation: *n* = 144). The iTED framework employed superpixel-based habitat determination using Gaussian mixture models on pretreatment dynamic contrast-enhanced MRI. Four predictive models were compared: clinical, conventional whole-tumor radiomics (C-radiomics), iTED, and combined (Clinical + iTED). The iTED model achieved higher discriminative performance compared with C-radiomics in both development (area under the curve [AUC]: 0.868 ± 0.068 vs. 0.730 ± 0.112) and external validation (AUC: 0.811 vs. 0.587) sets. The combined model further improved performance (development AUC: 0.908 ± 0.043; external AUC: 0.889). Habitat imaging-based iTED features achieved numerically higher performance than conventional radiomics in predicting Oncotype DX^®^ high-risk classification. These findings suggest the potential of iTED as a non-invasive imaging biomarker to support molecular testing in clinical decision-making.

## 1. Introduction

The Oncotype DX^®^ 21-gene recurrence score (RS) has become a standard tool for guiding adjuvant chemotherapy decisions in patients with estrogen receptor-positive, human epidermal growth factor receptor 2-negative (ER+/HER2−) early-stage breast cancer [1,2]. The TAILORx trial established that patients with RS > 25 are high-risk and derive significant benefit from adjuvant chemotherapy, whereas those with RS ≤ 25 (low/intermediate risk) can safely forgo chemotherapy when treated with endocrine therapy alone [1]. However, the Oncotype DX^®^ (ODX) assay has notable limitations: the test requires invasive tissue sampling from surgical specimens and involves substantial costs [3]. Additionally, tissue samples must be shipped to a centralized laboratory, resulting in considerable turnaround time [3]. These constraints highlight the need for non-invasive, cost-effective, and readily available complementary approaches for predicting breast cancer recurrence risk.

Intratumoral heterogeneity represents a fundamental characteristic of malignant tumors, reflecting diverse cell populations and microenvironmental conditions that influence treatment response and prognosis [4,5]. Habitat imaging has emerged as an analytical paradigm that conceptualizes tumors as complex ecosystems comprising multiple subregions (“habitats”) with distinct biological properties, such as perfusion, cellularity, and necrosis [6,7]. Habitat imaging employs unsupervised clustering algorithms to partition tumors based on imaging features, thereby capturing spatially distinct physiological microenvironments. Notably, studies have demonstrated the utility of habitat imaging in predicting pathological complete response to neoadjuvant chemotherapy in breast cancer [8,9,10], classifying clinically significant prostate cancer [11], and predicting recurrence in non-small cell lung cancer [12].

While previous imaging-based studies have attempted to predict ODX RS using conventional radiomics or deep learning approaches [13,14,15,16,17], these methods offer limited biological interpretability and insufficiently model spatial heterogeneity. Although habitat imaging has been applied to treatment response and outcome prediction across multiple cancer types [8,9,10,11,12], the application of habitat imaging to ODX RS prediction has not been explored. We hypothesize that habitat-based diversity metrics, by explicitly modeling the complexity of the tumor ecosystem, would provide complementary information about tumor heterogeneity associated with ODX RS classification compared to conventional whole-tumor radiomics approaches.

In this study, we developed the intratumoral tumor ecological diversity (iTED) framework, a habitat imaging approach for predicting ODX high-risk classification in ER+/HER2− breast cancer. The iTED framework quantifies tumor heterogeneity through superpixel-based habitat analysis with Gaussian mixture modeling on dynamic contrast-enhanced magnetic resonance imaging (DCE-MRI). This study aimed to develop and externally validate an iTED-based predictive model for ODX high-risk classification, compare performance across four models (clinical, C-radiomics, iTED, and combined), and identify the most important iTED features, thereby providing a non-invasive imaging-based tool to complement molecular testing and support clinical decision-making regarding the need for ODX testing.

## 2. Materials and Methods

### 2.1. Study Population

This retrospective multi-center study was approved by the institutional review boards at the participating institutions with an informed consent waiver. The study comprised a development set and an external validation set from two independent medical centers. Both sets included consecutive patients with ER+/HER2− invasive breast cancer who underwent ODX testing.

**Figure 1:**
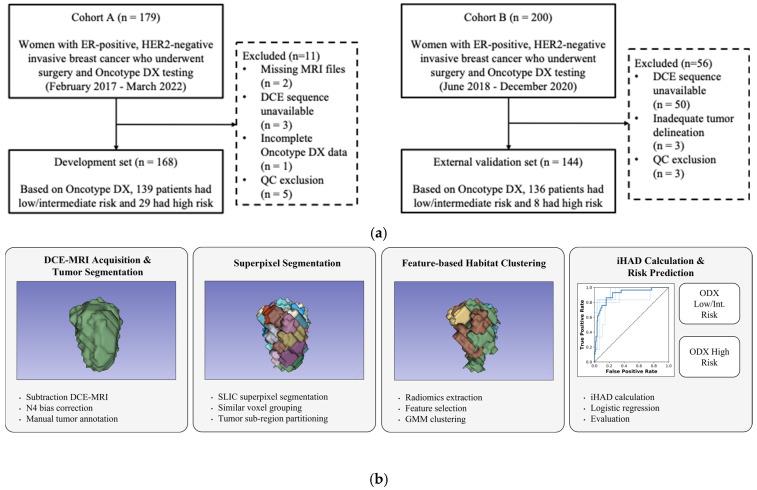
Patient selection and iTED workflow. (**a**) The flowchart shows patient selection for the development and external validation sets, including quality control filtering. Of the 179 patients initially assessed at center A, 173 met the initial criteria, and 168 were included after quality control. Of the 200 patients at center B, 147 met the initial criteria, and 144 were included after quality control. (**b**) The workflow shows the iTED calculation for ODX high-risk classification: tumor segmentation on DCE-MRI, SLIC superpixel segmentation, per-superpixel radiomics feature extraction, GMM-based habitat determination, and K-vector representation. ER = estrogen receptor; HER2 = human epidermal growth factor receptor 2; ODX = Oncotype DX^®^; DCE-MRI = dynamic contrast-enhanced magnetic resonance imaging; SLIC = simple linear iterative clustering; GMM = Gaussian mixture model; iTED = intratumoral tumor ecological diversity.

Inclusion criteria: (a) pathologically confirmed ER+/HER2− invasive breast cancer, (b) available ODX RS, (c) pretreatment dynamic contrast-enhanced magnetic resonance imaging (DCE-MRI), and (d) complete clinicopathological data. Exclusion criteria: (a) missing or incomplete DCE-MRI data, (b) incomplete ODX data, (c) inadequate tumor delineation, and (d) quality control failure in superpixel-based feature extraction (Figure 1a). Of the 179 patients initially assessed at center A (development set, February 2017–March 2022), 6 were excluded (2 with missing DCE-MRI data, 3 with incomplete DCE sequences, and 1 with incomplete ODX data). An additional 5 patients were excluded due to quality control failure during superpixel feature extraction, resulting in 168 included patients. Of the 200 patients initially assessed at center B (external validation set, June 2018–December 2020), 53 were excluded (50 with incomplete DCE sequences and 3 with inadequate tumor delineation), and 3 were further excluded due to quality control failure, resulting in 144 included patients. Among the excluded patients, all 11 from center A were classified as low/intermediate risk; the exclusions from center B included 7 high-risk patients, reflecting the imaging-based (rather than risk-based) nature of the exclusion criteria.

The primary objective was to develop and validate an iTED-based model for classifying ODX as high risk. Secondary objectives included comparing the performance of the iTED-based model against clinical variables and conventional radiomics and identifying the most important features in the iTED model.

### 2.2. Image Acquisition and Preprocessing

Pretreatment, all patients underwent DCE-MRI using 3T scanners at both institutions; detailed acquisition parameters are provided in Table A1. Image preprocessing included reorientation to a standardized LPS (left posterior–superior) coordinate system, resampling to 1 mm^3^ isotropic voxels using linear interpolation, N4 bias field correction [18], and Nyul intensity normalization [19] with landmarks computed from the development set. The second post-contrast subtraction image was used for subsequent analysis.

### 2.3. Intratumoral Tumor Ecological Diversity (iTED) Framework

The iTED framework quantifies tumor heterogeneity by analyzing spatial variations in imaging phenotypes (Figure 1b). The framework consists of three sequential steps: (1) tumor segmentation, (2) superpixel segmentation, and (3) habitat-based feature extraction and K-vector representation. For comparison, conventional whole-tumor radiomics (C-radiomics) features were also extracted from the entire tumor volume.

Tumor segmentation was performed using an in-house annotation tool on the subtraction of the DCE-MRI data by a radiology resident (3 years of experience). All segmentations were reviewed and finalized by a board-certified breast radiologist (>10 years of experience). The tumor was annotated using three-dimensional (3D) volumetric masks encompassing the entire tumor across all slices.

Superpixel segmentation was performed using simple linear iterative clustering (SLIC) on the second post-contrast subtraction image [20]. A fixed allocation of 50 superpixels per tumor was applied regardless of tumor volume, with a compactness parameter of 1 and sigma of 3.0. A sensitivity analysis across different superpixel counts confirmed the robustness of this choice (Table A2). The SLIC algorithm was applied in 3D to the masked tumor region, generating spatially coherent subregions that respect both spatial proximity and intensity similarity. Superpixels extending beyond the tumor boundary were excluded from analysis.

For the iTED calculation, radiomics features were extracted from each superpixel region using PyRadiomics (version 3.0.1) [21] following the Image Biomarker Standardization Initiative (IBSI) guidelines [22] with a fixed bin width of 50. A total of 93 features were extracted per superpixel across six feature classes: first-order statistics (18 features), gray-level co-occurrence matrix (GLCM; 24 features), gray-level dependence matrix (GLDM; 14 features), gray-level run-length matrix (GLRLM; 16 features), gray-level size-zone matrix (GLSZM; 16 features), and neighborhood gray-tone difference matrix (NGTDM; 5 features). Shape features were excluded as superpixel shapes are determined by the SLIC algorithm rather than biological properties. For each radiomics feature, a Gaussian mixture model (GMM) was fitted to the feature distribution across all superpixels within the tumor, with the optimal number of components (K = 1–10) determined by the Akaike information criterion (AIC). The optimal K value for each radiomics feature served as the corresponding iTED feature, yielding a 93-dimensional K-vector for each patient. For C-radiomics, the same 93 features were extracted from the entire tumor volume as a single region of interest using the same PyRadiomics settings.

### 2.4. Model Development and Validation

Four predictive models were developed and compared: (1) a clinical model using six clinical variables (age, tumor size, Ki-67 index, nuclear grade, histologic grade, and progesterone receptor status); (2) a C-radiomics model using conventional whole-tumor radiomics features; (3) an iTED model using K-vector features; and (4) a combined model integrating clinical variables with selected iTED features. Progesterone receptor status was included because despite partial overlap with the ODX gene panel, it is a routinely available immunohistochemical marker that provides independent clinical information for risk stratification.

For the C-radiomics and iTED models, L1-regularized logistic regression (LASSO) was used for feature selection on the full development set to address the low events per variable ratio (29 high-risk events/93 features). The L1 regularization strength was determined via 3-fold cross-validation within the development set, and features with non-zero coefficients were selected for downstream modeling. This yielded 20 features for C-radiomics and 14 features for iTED. All models used L2 regularization with logistic regression. Regularization strength (C) and class weights were optimized via grid search on cross-validated AUC: clinical (C = 1.0, class weight = 1:5), C-radiomics (C = 1.0, balanced), iTED (C = 0.1, class weight = 1:3), and combined (C = 0.1, class weight = 1:3).

Model development employed stratified 5-fold cross-validation. For each fold, the development set was split into 80% for training and 20% for testing; each patient appeared in the test set exactly once across the five folds. Two missing progesterone receptor values were imputed with median values. All features were z-score normalized using statistics from the training set within each cross-validation fold. Classification thresholds were determined using Youden’s J statistic.

To address class imbalance between the high-risk and low/intermediate-risk groups, class weighting was applied. Model performance was evaluated using the area under the curve (AUC) as the primary metric, along with sensitivity and specificity. The final model was trained on the entire development set and evaluated on the independent external validation set, which served as the primary measure of model performance. For the external validation set, 95% confidence intervals (CIs) for AUC, sensitivity, and specificity were calculated using bootstrap resampling (2000 iterations).

### 2.5. Histopathological Analysis

Representative histopathological specimens were reviewed by a board-certified pathologist. Hematoxylin and eosin-stained sections were examined at low power (×20) and high power (×200) magnification to assess the tumor growth patterns, cellular architecture, nuclear grade features, and stromal response characteristics.

### 2.6. Statistical Analysis

Baseline clinicopathologic characteristics were compared between sets using independent *t*-tests for continuous variables and chi-square or Fisher’s exact tests for categorical variables. Comparisons between risk groups were performed using Mann–Whitney U tests for continuous variables and chi-square tests for categorical variables. Pairwise comparisons of AUC between models were performed using the DeLong test. Statistical significance was defined as two-sided *p* < 0.05. All analyses were performed using Python (version 3.7.4) with the scikit-learn (version 0.21.3), scipy (version 1.7.3), and statsmodels (version 0.13.5) libraries. Feature importance was assessed using SHAP (SHapley Additive exPlanations, version 0.42.1) analysis [23] with a linear explainer on the full development set training data. Decision curve analysis was performed to assess the net clinical benefit of predictive models across a range of threshold probabilities [24].

## 3. Results

### 3.1. Patient Characteristics

A total of 320 patients met the initial inclusion criteria (development set: *n* = 173; external validation set: *n* = 147). After quality control filtering, 312 patients were included in the final analysis (development: *n* = 168; external: *n* = 144) (Table 1). The two sets showed no significant differences in age (*p* = 0.210), menopausal status (*p* = 0.367), clinical tumor size (*p* = 0.085), pathologic tumor size (*p* = 0.295), tumor type (*p* = 0.532), or metastatic lymph node status (*p* = 0.165) (all *p* > 0.05). However, the sets differed significantly in nuclear grade (*p* < 0.001), histological grade (*p* < 0.001), lymphovascular invasion (*p* < 0.001), Ki-67 index (*p* < 0.001), and progesterone receptor status (*p* < 0.001).

When stratified by the TAILORx criteria, 275 patients (88.1%) were classified as low/intermediate risk (RS ≤ 25) and 37 patients (11.9%) as high risk (RS >25) (development: 139 low/intermediate-risk and 29 high-risk; external: 136 low/intermediate-risk and 8 high-risk). During a mean follow-up of 47.3 ± 14.0 months, locoregional recurrence occurred in five patients (1.6%) and distant metastasis in three patients (1.0%). Distant metastasis was significantly more frequent in the high-risk group compared to the low/intermediate-risk group (5.4% vs. 0.4%; *p* = 0.038) (Table 2).

### 3.2. Feature Selection

Feature selection via L1-regularized LASSO was applied independently to the C-radiomics and iTED features in the development set. For C-radiomics, starting with 93 whole-tumor radiomics features, LASSO selected 20 features with non-zero coefficients. For iTED, beginning with 93 K-vector features, LASSO selected 14 features with non-zero coefficients (Table A3). Among the 14 selected iTED features, four first-order features were most represented (interquartile range, range, median, and robust mean absolute deviation), followed by three GLCM features (correlation, difference entropy, and inverse difference moment normalized), three NGTDM features (contrast, coarseness, and busyness), three GLRLM features (short run low gray level emphasis, gray level non-uniformity, and short run high gray level emphasis), and one GLSZM feature (zone percentage). The top-ranked features by absolute LASSO coefficient magnitude were GLCM Correlation, GLCM Difference Entropy, and NGTDM Contrast.

### 3.3. Predictive Performance

**Figure 2:**
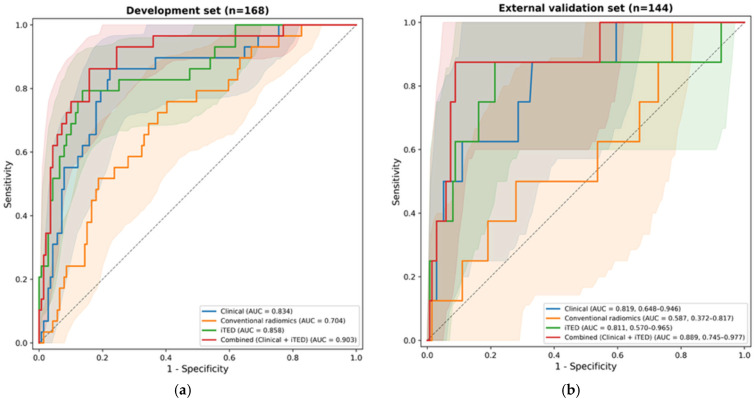
Receiver operating characteristic (ROC) curve analysis with 95% bootstrap confidence intervals (shaded bands, 2000 iterations). ROC curves for the (**a**) development set (5-fold cross-validation) and (**b**) external validation set. Four models are compared: clinical (blue), conventional radiomics (orange), iTED (green), and combined (Clinical + iTED, red). AUC (area under the curve) values with mean ± standard deviation across folds are shown for the development set.

In the development set, the iTED model achieved higher discriminative performance compared to C-radiomics and the clinical model (Figure 2a; Table 3). The iTED model achieved an AUC of 0.868 ± 0.068, compared to 0.730 ± 0.112 for C-radiomics and 0.821 ± 0.083 for the clinical model. The combined model (Clinical + iTED) achieved the highest development AUC of 0.908 ± 0.043. The iTED model demonstrated a sensitivity of 0.793 ± 0.124 and specificity of 0.813 ± 0.099.

In the external validation set (Figure 2b; Table 3), the iTED model achieved an AUC of 0.811 (95% CI: 0.570–0.965) compared to 0.587 (95% CI: 0.372–0.817) for C-radiomics and 0.819 (95% CI: 0.648–0.946) for the clinical model. The combined model achieved the highest external validation AUC of 0.889 (95% CI: 0.745–0.977), with a sensitivity of 0.875 and specificity of 0.853. The DeLong test comparing AUCs in the external validation set showed a statistically significant difference between the clinical and C-radiomics models (*p* = 0.006). Although the iTED model showed a substantially higher AUC than C-radiomics (0.811 vs. 0.587), this difference did not reach statistical significance (*p* = 0.232). The iTED model achieved an AUC comparable to the clinical model (*p* = 0.951). The combined model yielded the highest AUC (0.889), although the difference from the clinical model did not reach statistical significance (*p* = 0.480).

### 3.4. Subgroup and Calibration Analysis

**Figure 3:**
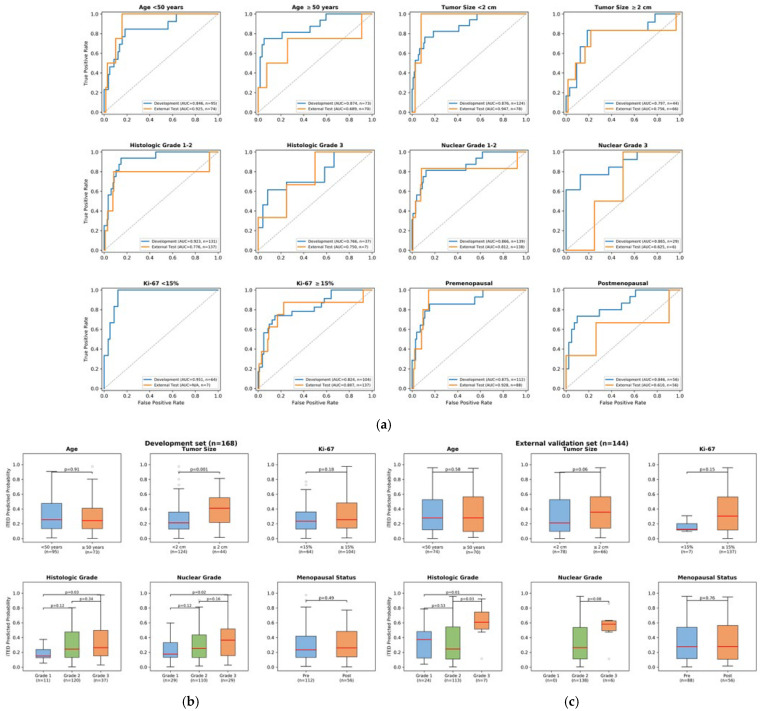
Subgroup analysis of the iTED model. (**a**) Subgroup-specific ROC curves for the iTED model within clinical subgroups for both the development and external validation sets. (**b**) Predicted probability box plots stratified by clinical subgroups for the development set. (**c**) Predicted probability box plots stratified by clinical subgroups for the external validation set.

Subgroup-specific receiver operating characteristic (ROC) analysis showed that the iTED model maintained discriminative ability across age groups, tumor size categories, histologic grades, and menopausal status in both development and external validation sets (Figure 3a). The iTED model predicted probabilities showed consistent discrimination between the high-risk and low/intermediate-risk groups across all examined subgroups in both sets (Figure 3b,c).

**Figure A1:**
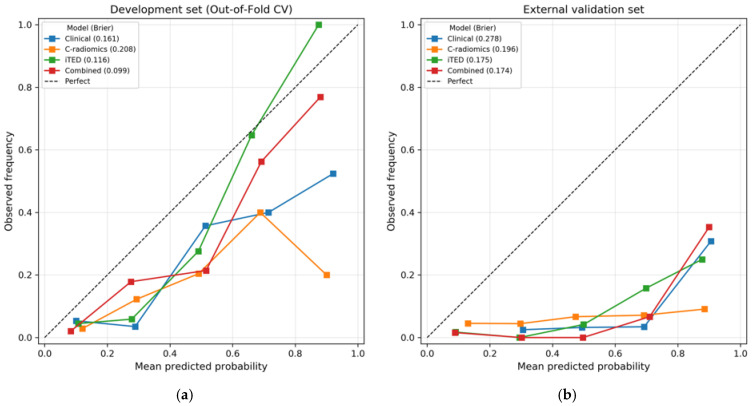
Calibration analysis. Calibration curves comparing the predicted probabilities versus the observed frequencies for four models. (**a**) Development set. (**b**) External validation set. Brier scores are reported for each model.

Calibration analysis using out-of-fold predictions in the development set showed that the combined and iTED models achieved Brier scores (0.099 and 0.116, respectively) lower than the null model (0.143), whereas clinical (0.161) and C-radiomics (0.208) exceeded the null model Brier score (Figure A1, panel a). In the external validation set, all models exceeded the null Brier score (0.052; combined: 0.174, iTED: 0.175, C-radiomics: 0.196, clinical: 0.278) (Figure A1, panel b).

### 3.5. Feature Importance

**Figure 4:**
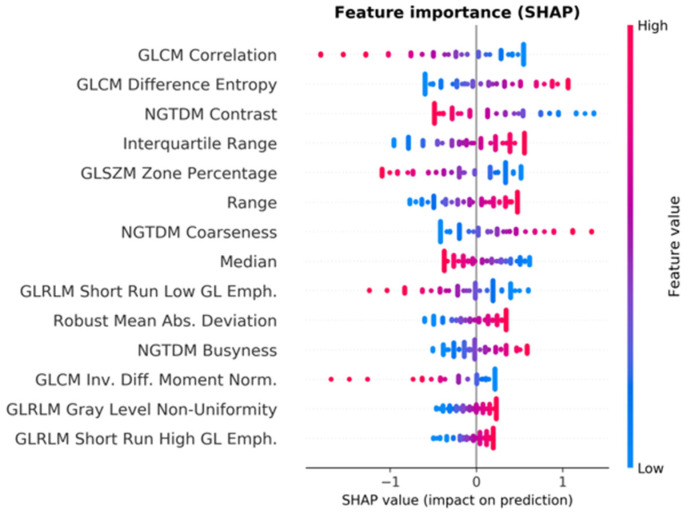
SHAP (SHapley Additive exPlanations) beeswarm plot showing the contribution of 14 selected iTED features to model predictions. Features are ranked by mean absolute SHAP value. Each dot represents one patient; color indicates the feature value (red = high, blue = low).

SHAP analysis was performed on the iTED model trained on the full development set to quantify individual feature contributions to the predicted probability of high-risk classification (Figure 4). The three most important features by mean absolute SHAP value were GLCM correlation (0.523), GLCM difference entropy (0.511), and NGTDM contrast (0.441). At the category level, first-order statistics collectively contributed the largest impact (cumulative mean |SHAP| = 1.391), followed by GLCM (1.260), NGTDM (1.048), GLRLM (0.646), and GLSZM (0.423). Directionality analysis revealed two groups: higher K values in GLCM difference entropy, first-order interquartile range (IQR), range, and robust mean absolute deviation (rMAD), NGTDM coarseness and busyness, and GLRLM gray level non-uniformity (GLNU) and short run high gray level emphasis (SRHGLE) were positively associated with high-risk prediction, whereas higher K values in GLCM correlation and inverse difference moment normalized (IDMN), NGTDM contrast, GLSZM zone percentage, first-order median, and GLRLM short run low gray level emphasis (SRLGLE) were negatively associated.

### 3.6. Representative Cases

**Figure 5:**
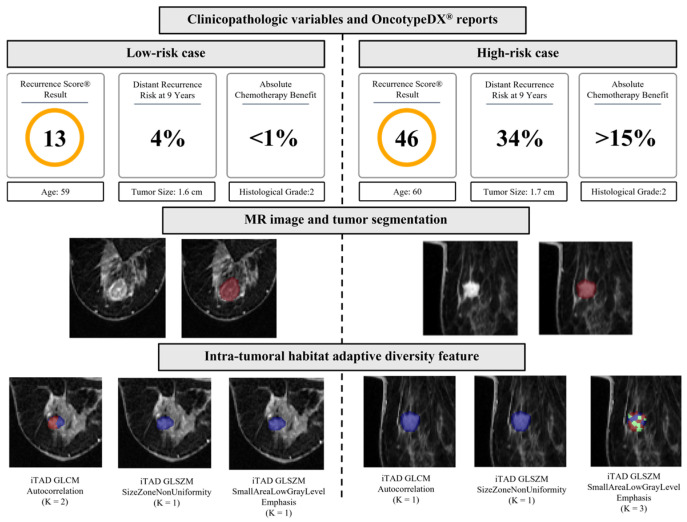
Representative cases with distinct habitat clustering patterns. Two patients with similar clinicopathological features but different ODX recurrence scores (left: RS = 13, right: RS = 46) demonstrated distinct habitat clustering patterns across three representative iTED features: NGTDM contrast, GLRLM GLNU, and NGTDM busyness. Habitat maps for all 14 selected iTED features across additional cases are shown in Figure A2.

**Figure A2:**
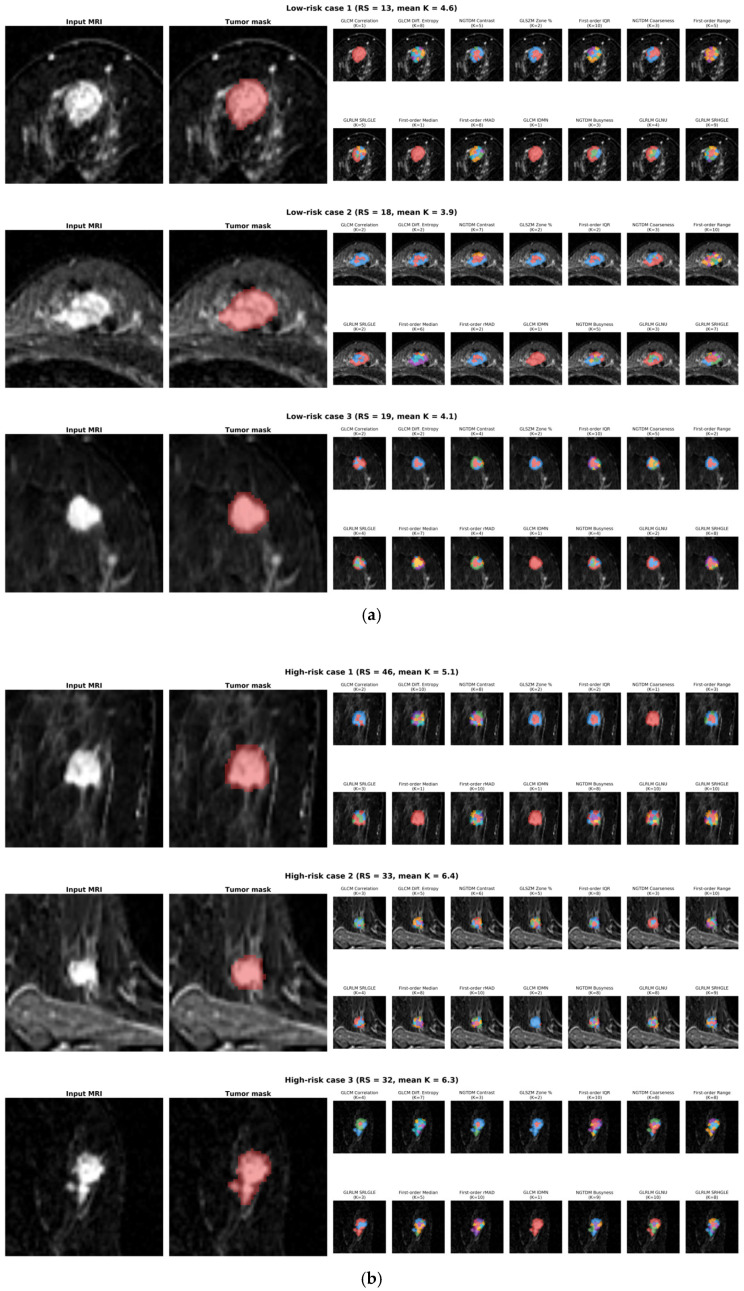
Representative iTED habitat maps for 14 selected features. (**a**) Three low-risk cases and (**b**) three high-risk cases showing habitat clustering patterns across all 14 selected iTED features, demonstrating distinct habitat diversity between risk groups.

Figure 5 shows two patients with similar clinicopathological characteristics (age 59 vs. 60 years, tumor size 1.6 vs. 1.7 cm, histological grade 2) but different Oncotype DX^®^ risk classifications (RS = 13 vs. RS = 46). The high-risk patient showed higher K values in most iTED features (mean K = 5.1 vs. 4.6), particularly in GLCM difference entropy (10 vs. 8), NGTDM busyness (8 vs. 3), and GLRLM GLNU (10 vs. 4). Habitat maps for all 14 features across additional representative cases are presented in Figure A2.

**Figure 6:**
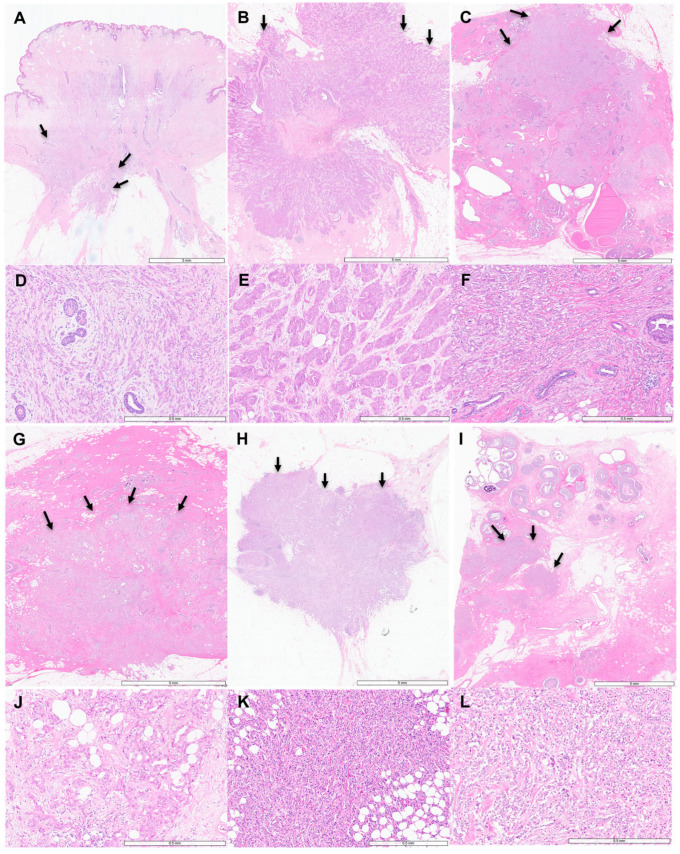
Representative hematoxylin and eosin-stained histopathologic images of breast cancer cases from the external validation set. Low-risk cases (RS ≤ 25): ((**A**–**C**), ×20) Low-power views demonstrate invasive carcinomas with distinct growth patterns, including an infiltrative carcinoma located in the subareolar region beneath the nipple–areolar complex, a representative spiculated (stellate) tumor mass with malignant nests radiating into the adjacent mammary adipose tissue, and a cohesive solid tumor showing an irregular invasive front at the interface with the surrounding fatty stroma (arrows). ((**D**–**F**), ×200) High-power views demonstrate infiltrative breast carcinoma composed of small nests, cords, and cohesive clusters of neoplastic cells, accompanied by variable nuclear pleomorphism and a prominent desmoplastic stromal response, with focal irregular tumor clusters and mild chronic inflammatory cell infiltration. High-risk cases (RS > 25): ((**G**–**I**), ×20) Low-power views demonstrate invasive carcinomas with expansile solid growth patterns, including a broad sheet-like tumor mass with an irregular pushing invasive front, a large lobulated tumor with well-defined margins extending into the surrounding parenchyma, and a solid invasive carcinoma with associated stromal desmoplasia (arrows). ((**J**–**L**), ×200) High-power views demonstrate solid sheets and confluent nests of neoplastic cells with high-grade nuclear features, marked nuclear pleomorphism, prominent nucleoli, and increased mitotic activity, with focal comedo-type necrosis and minimal intervening stroma.

Representative histopathological images demonstrated distinct morphologic patterns between risk groups (Figure 6). Low-risk cases (Figure 6A–F) exhibited predominantly infiltrative growth patterns with irregular tumor–stroma interfaces and variable spatial distribution of tumor cell density. High-risk cases (Figure 6G–L) more frequently demonstrated solid growth patterns with compact cellular aggregation and high-grade nuclear features. Despite similar overall cellularity, the spatial organization differed markedly: low-risk tumors exhibited dispersed, infiltrative extension, whereas high-risk tumors formed cohesive solid masses with a repetitive, clustered arrangement.

## 4. Related Work

Conventional radiomics extracts quantitative features from an entire tumor region, implicitly treating the lesion as a homogeneous entity. However, solid tumors are spatially heterogeneous, comprising sub-regions that differ in vascularity, cellularity, and necrosis [4,5]. Habitat imaging addresses this limitation by partitioning the tumor into spatially distinct sub-regions—termed habitats—through the clustering of multiparametric imaging maps [6,7], an approach recently characterized as “Radiomics++” for its capacity to decode intratumoral heterogeneity beyond whole-tumor radiomics [25].

In breast cancer, Shi et al. [8] applied SLIC-based habitat partitioning with Gaussian mixture clustering of MRI radiomics to derive intratumoral ecological diversity features for predicting pathological complete response (pCR) to neoadjuvant chemotherapy. Subsequent studies extended habitat imaging to predict pCR using intravoxel incoherent motion MRI [9] and assess recurrence-free survival via perfusion-based spatial heterogeneity mapping [10]. Beyond breast cancer, habitat imaging has been applied to prostate [11] and lung cancer [12]. More recently, Chen et al. [26] employed habitat-based MRI radiomics to classify HER2 expression status, and Huang et al. [27] integrated longitudinal MRI habitat features with transcriptomics to predict pCR. However, these applications have been confined to treatment response or receptor status prediction, and no prior work has extended habitat imaging to genomically defined recurrence risk as measured by multigene assays such as Oncotype DX.

Several studies have investigated MRI-based approaches to predict ODX recurrence scores non-invasively. In early correlational work, Li et al. [16] first demonstrated significant associations between MRI radiomics signatures and multigene assay recurrence scores, including Oncotype DX. Subsequent efforts broadened the methodological repertoire, encompassing qualitative imaging features [14], DCE-MRI-based machine learning models [17], and radiomics with various classifiers [13], while Ha et al. [15] applied convolutional neural networks to ODX recurrence score prediction as a proof-of-concept. More recent work has explored feature enrichment strategies: Chiacchiaretta et al. [28] incorporated peritumoral MRI radiomics, and Zhang et al. [29] developed a multimodal deep learning model integrating multi-sequence MRI with clinicopathologic features. A systematic review by Kim et al. [30] summarized these efforts, highlighting that multiparametric MRI approaches yielded the highest performance. Nevertheless, all of these studies extracted features from the whole tumor volume, providing a global summary that does not explicitly resolve habitat-level heterogeneity within the lesion.

Taken together, prior work has pursued two parallel directions: habitat imaging for other clinical endpoints, and whole-tumor radiomics for ODX recurrence score prediction. However, their intersection—habitat-based characterization of genomically defined recurrence risk—remains unexplored. To our knowledge, the present study is the first to apply habitat imaging to the prediction of ODX-defined genomic recurrence risk and to validate the approach on an independent external cohort.

## 5. Discussion

This study developed and externally validated an MRI-based iTED framework for predicting ODX high-risk classification in patients with ER+/HER2− breast cancer. Our findings demonstrate that the iTED model—despite relying solely on imaging—achieved discriminative performance comparable to a clinical model built on established clinicopathological variables, including the immunohistochemical markers PR and Ki-67 (AUCs of 0.868 vs. 0.821 in development and 0.811 vs. 0.819 in external validation; DeLong *p* = 0.951 in external validation), and numerically higher than conventional radiomics (AUCs of 0.730 and 0.587, respectively) while maintaining robust sensitivity across institutions. The combined model (Clinical + iTED) achieved the highest AUCs (0.908 and 0.889), although the improvement over the clinical model was not statistically significant (DeLong *p* = 0.480). Among the selected features, GLCM correlation emerged as the most important predictor, followed by GLCM difference entropy and NGTDM contrast.

Previous imaging-based approaches to predict ODX recurrence scores have primarily relied on conventional radiomics or deep learning methods. These approaches share important limitations, including whole-tumor feature averaging, which obscures spatial heterogeneity, and limited biological interpretability. Our iTED approach addresses these limitations by explicitly modeling intratumoral spatial complexity through habitat-based diversity metrics. The higher external validation performance of the iTED model (AUC 0.811) compared to C-radiomics (AUC 0.587) suggests that spatially resolved habitat characterization provides clinically relevant information beyond conventional approaches. Moreover, the comparable AUC between the iTED and clinical models in external validation (DeLong *p* = 0.951) suggests that habitat-based imaging features can approximate the discriminative ability of clinicopathological variables. The poor external generalizability of C-radiomics likely reflects the vulnerability of whole-tumor models to interinstitutional variations in MRI acquisition and reconstruction, whereas the iTED approach appears more robust to such variations through its relative quantification of habitat diversity.

The higher performance of the iTED approach likely reflects its ability to capture biologically meaningful patterns of intratumoral heterogeneity that correlate with genomic diversity. GLCM correlation, the most important predictor in our model (mean |SHAP| = 0.523), measures local correlation in voxel intensity patterns; higher K values indicate greater diversity in spatial correlation patterns within the tumor, suggesting more heterogeneous tissue organization. GLCM difference entropy (mean |SHAP| = 0.511) quantifies the entropy of intensity differences between neighboring voxels, with higher habitat diversity reflecting increased microstructural complexity. NGTDM contrast (mean |SHAP| = 0.441) captures intensity differences between a voxel and its neighborhood, with higher K values in high-risk tumors suggesting greater contrast diversity across habitat regions. These patterns align with the current biological understanding that genomically high-risk tumors often exhibit altered vascular architecture, hypoxic microenvironments, and disrupted cellular organization. Notably, the iTED-based heterogeneity does not merely reflect tumor burden or cellularity, but rather the spatial organization and growth behavior of tumor cells, as confirmed by histopathologic analysis (Figure 6).

**Figure A3:**
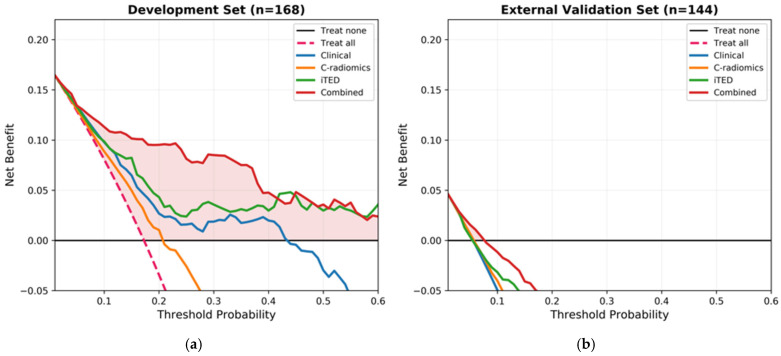
Decision curve analysis. Net benefit curves for four models compared with treat-all (dashed) and treat-none (solid) strategies. (**a**) Development set (*n* = 168). (**b**) External validation set (*n* = 144).

From a clinical perspective, the iTED framework offers several potential advantages as a non-invasive triage tool to guide ODX testing decisions. First, the iTED framework utilizes routinely acquired DCE-MRI sequences, requiring no additional imaging protocols or patient burden. Second, the consistently high sensitivity across sets (0.793 in development, 0.875 in external validation) suggests that the model is particularly useful for identifying high-risk patients who would benefit from ODX testing, ensuring that high-risk patients are not missed. The combined model further improved discrimination, providing a potential two-stage screening approach: an initial iTED assessment followed by ODX testing for patients identified as high risk. Third, the framework delivers immediate results, avoiding turnaround times of several weeks for centralized laboratory analysis. Decision curve analysis further supported these findings, with the Combined model demonstrating positive net benefit across a range of threshold probabilities in the development set (Figure A3).

This study has several limitations. First, the retrospective design and inclusion of only two institutions limit generalizability, as variations in MRI protocols and patient populations may affect model performance. The relatively small number of high-risk cases in the external validation set (*n* = 8, 5.6%) further restricted our ability to assess model robustness across diverse patient subgroups and resulted in wide confidence intervals, underscoring the need for validation in larger sets. However, this distribution reflects real-world prevalence in ER+/HER2− breast cancer. Second, tumor segmentation was performed by a single annotator without interobserver variability assessment, and future studies should evaluate reproducibility across multiple readers. Third, although calibration was assessed via Brier scores and calibration curves, the model may require site-specific recalibration before clinical deployment to account for differences in disease prevalence and imaging protocols across institutions. Notably, all models exceeded the null Brier score in external validation, likely reflecting the combined effects of low event rate and class weighting designed to optimize sensitivity. Fourth, we did not directly assess the predictive value for clinical outcomes, such as distant recurrence or treatment benefit. Therefore, prospective validation with long-term outcome data is needed to establish clinical utility. Fifth, the classification threshold was determined using Youden’s J statistic, which optimizes the balance between sensitivity and specificity but does not account for the differential clinical costs of false-negative versus false-positive classifications. In settings where the consequences of missing a high-risk patient are substantially greater, a cost-sensitive threshold may be more appropriate and warrants investigation in future studies.

Future research should prioritize validation in larger, multi-center sets and prospective studies evaluating the cost-effectiveness of iTED-guided selective ODX testing. Additionally, extending the iTED framework to other molecular assays and solid tumor types, and integrating with multi-omics data, may further broaden clinical applicability.

## 6. Conclusions

This study demonstrates that MRI-based intratumoral tumor ecological diversity features can predict ODX high-risk classification in ER+/HER2− breast cancer. The iTED model achieved numerically higher discriminative performance than conventional radiomics in both sets and comparable performance to clinical variables. Combining iTED with clinical features yielded the numerically highest AUC, although the improvement over clinical variables alone was not statistically significant. The iTED framework offers a non-invasive approach that, through its consistent sensitivity across institutions, may serve as a triage tool to complement molecular testing in clinical decision-making.

## Figures and Tables

**Table 1:** Clinicopathological characteristics of patients with breast cancer who underwent Oncotype DX^®^.

Variable	Development Set (*n* = 168)	External Validation Set (*n* = 144)	*p*-Value
Age (mean ± SD, years)	49.07 ± 8.58	50.77 ± 9.88	0.210
Menopausal status			0.367
Pre-menopause	112 (66.67)	88 (61.11)	
Post-menopause	56 (33.33)	56 (38.89)	
Clinical tumor size (mean ± SD, cm)	1.68 ± 0.92	1.97 ± 1.29	0.085
Pathologic tumor size (mean ± SD, cm)	1.76 ± 0.68	1.91 ± 0.86	0.295
Type of tumor (n, %)			0.532
Invasive ductal carcinoma	149 (88.69)	134 (93.06)	
Invasive lobular carcinoma	17 (10.12)	8 (5.56)	
Mixed IDC and ILC	1 (0.60)	1 (0.69)	
Mucinous carcinoma	1 (0.60)	1 (0.69)	
Nuclear grade (n, %)			<0.001
Grade 1	29 (17.26)	0 (0.00)	
Grade 2	110 (65.48)	138 (95.83)	
Grade 3	29 (17.26)	6 (4.17)	
Histological grade (n, %)			<0.001
Grade 1	11 (6.55)	24 (16.67)	
Grade 2	120 (71.43)	113 (78.47)	
Grade 3	37 (22.02)	7 (4.86)	
Lymphovascular invasion (n, %)	72 (42.86)	35 (24.31)	<0.001
Metastatic axillary LN (n, %)	14 (8.33)	20 (13.89)	0.165
No. of metastatic LN (mean ± SD)	0.10 ± 0.35	0.17 ± 0.46	0.106
No. of removed LN (mean ± SD)	3.91 ± 2.23	4.76 ± 3.27	0.052
ER positive (n, %)	168 (100.00)	144 (100.00)	N/A
PR positive (n, %)	155 (92.26)	144 (100.00)	<0.001
Ki-67 index (mean ± SD, %)	21.19 ± 14.10	35.21 ± 19.85	<0.001
Low (<15%)	64 (38.10)	7 (4.86)	
High (≥15%)	104 (61.90)	137 (95.14)	

SD = standard deviation; LN = lymph node; ER = estrogen receptor; PR = progesterone receptor; N/A = not applicable.

**Table 2:** Clinical outcomes according to Oncotype DX^®^ risk stratification.

Variable	Total (*n* = 312)	Low/Intermediate (*n* = 275)	High-Risk (*n* = 37)	*p*-Value
Mean RS (±SD)	16.2 ± 7.8	14.1 ± 5.3	31.7 ± 5.1	<0.001
Development set	168	139	29	
External validation set	144	136	8	
Follow-up (mean ± SD, months)	47.3 ± 14.0	47.8 ± 14.1	44.1 ± 12.9	0.075
Distant metastasis (n, %)	3 (1.0%)	1 (0.4%)	2 (5.4%)	0.038

RS = recurrence score; SD = standard deviation.

**Table 3:** Prediction performance of Oncotype DX^®^ recurrence score risk stratification.

Model	Metric	Development Set (*n* = 168)	External Validation Set (*n* = 144)
Clinical	AUC	0.821 ± 0.083	0.819 (0.648–0.946)
	Sensitivity	0.720 ± 0.142	0.875 (0.571–1.000)
	Specificity	0.821 ± 0.108	0.618 (0.536–0.697)
C-radiomics	AUC	0.730 ± 0.112	0.587 (0.372–0.817)
	Sensitivity	0.627 ± 0.155	0.500 (0.143–0.889)
	Specificity	0.655 ± 0.071	0.581 (0.496–0.664)
iTED	AUC	0.868 ± 0.068	0.811 (0.570–0.965)
	Sensitivity	0.793 ± 0.124	0.875 (0.600–1.000)
	Specificity	0.813 ± 0.099	0.728 (0.652–0.799)
Combined	AUC	0.908 ± 0.043	0.889 (0.745–0.977)
	Sensitivity	0.660 ± 0.254	0.875 (0.600–1.000)
	Specificity	0.900 ± 0.083	0.853 (0.794–0.911)

Development set: mean ± SD from 5-fold cross-validation. External validation set: point estimates from the final model trained on the entire development set; 95% CI for AUC, sensitivity, and specificity via bootstrap (*n* = 2000). Classification threshold determined by Youden’s J statistic.

## Data Availability

The data presented in this study are available on request from the corresponding author. The data are not publicly available due to patient privacy.

## Abbreviations

The following abbreviations are used in this manuscript:

AIC	Akaike information criterion
AUC	Area under the curve
BIC	Bayesian information criterion
C-radiomics	Conventional whole-tumor radiomics
CI	Confidence interval
DCE-MRI	Dynamic contrast-enhanced magnetic resonance imaging
ER	Estrogen receptor
GLCM	Gray-level co-occurrence matrix
GLDM	Gray-level dependence matrix
GLRLM	Gray-level run length matrix
GLSZM	Gray-level size zone matrix
GMM	Gaussian mixture model
HER2	Human epidermal growth factor receptor 2
IBSI	Image Biomarker Standardization Initiative
iTED	Intratumoral tumor ecological diversity
NGTDM	Neighboring gray tone difference matrix
NPV	Negative predictive value
ODX	Oncotype DX
PPV	Positive predictive value
ROC	Receiver operating characteristic
RS	Recurrence score
SHAP	SHapley Additive exPlanations
SLIC	Simple linear iterative clustering

## Appendix A

### Appendix A.1. Supplementary Tables

**Table A1:** MRI acquisition parameters for the development and external validation sets.

Parameter	Development (KNUCH)	External Validation (PNU)
Manufacturer	GE Medical Systems	Siemens
Model	Discovery MR750	Skyra_TC
Field strength	3 T	3 T
TR (ms)	4.7 ± 0.8 (4.1–6.7)	5.5 ± 0.1 (4.0–5.5)
TE (ms)	1.8 ± 0.0 (1.7–1.8)	2.5 ± 0.1 (1.5–2.5)
Flip angle (°)	12.4 ± 2.5 (10.0–15.0)	12.0 ± 0.3 (10.0–12.0)
Matrix	512 × 512	384 × 384
FOV (mm)	323.3 × 323.3	352.2 × 352.0
Slice thickness (mm)	2.0 ± 0.0	0.9 ± 0.0

TR = repetition time; TE = echo time; FOV = field of view; T = Tesla. Data are mean ± SD (range).

**Table A2:** Sensitivity analysis of superpixel count for the iTED model.

Superpixels (*n*)	Dev AUC (Mean ± SD)	Ext AUC (95% CI)	Ext Sensitivity
25	0.759 ± 0.067	0.562 (0.308–0.796)	0.375
50 *	0.868 ± 0.068	0.811 (0.570–0.965)	0.875
100	0.848 ± 0.037	0.623 (0.403–0.837)	0.500

* Primary configuration. All models: L2-penalized logistic regression, LASSO feature selection, 5-fold cross-validation, bootstrap 95% CI (*n* = 2000).

**Table A3:** Features selected for the conventional radiomics and iTED models.

Rank	Feature	Feature Class	Coefficient
C-radiomics model (20 features)			
1	Maximum	First-order	−1.1323
2	Kurtosis	First-order	−0.8189
3	Size Zone Non-Uniformity	GLSZM	0.6921
4	Zone Percentage	GLSZM	0.6126
5	Range	First-order	−0.5155
6	Median	First-order	0.5126
7	Cluster Prominence	GLCM	−0.4555
8	Run Variance	GLRLM	−0.4097
9	Strength	NGTDM	−0.3917
10	Informational Measure of Correlation 2	GLCM	−0.3663
11	Informational Measure of Correlation 1	GLCM	0.3311
12	Joint Energy	GLCM	0.2766
13	Contrast	NGTDM	−0.2274
14	Complexity	NGTDM	0.1579
15	Large Area Low Gray Level Emphasis	GLSZM	0.1470
16	Maximal Correlation Coefficient	GLCM	−0.1371
17	Zone Entropy	GLSZM	−0.1189
18	Low Gray Level Run Emphasis	GLRLM	0.1074
19	Skewness	First-order	0.0566
20	Gray Level Non-Uniformity Normalized	GLSZM	0.0375
iTED model (14 features)			
1	Correlation	GLCM	−0.6415
2	Difference Entropy	GLCM	0.5865
3	Contrast	NGTDM	−0.5271
4	Zone Percentage	GLSZM	−0.5094
5	Interquartile Range	First-order	0.4964
6	Coarseness	NGTDM	0.4546
7	Range	First-order	0.4172
8	Short Run Low Gray Level Emphasis	GLRLM	−0.3984
9	Median	First-order	−0.3498
10	Robust Mean Absolute Deviation	First-order	0.3210
11	Inverse Difference Moment Normalized	GLCM	−0.3194
12	Busyness	NGTDM	0.2987
13	Gray Level Non-Uniformity	GLRLM	0.2176
14	Short Run High Gray Level Emphasis	GLRLM	0.1865

Feature selection: L1-penalized logistic regression (LASSO) with 3-fold CV on the development set. Coefficients from the final L2-regularized model. Features ranked by absolute coefficient value.
